# Infantile hypertrophic pyloric stenosis, the cause of non‐bilious vomiting of a 3‐day‐old male infant with situs inversus totalis: A case report

**DOI:** 10.1002/ccr3.6238

**Published:** 2022-08-09

**Authors:** Ali Samady Khanghah, Khashayar Atqiaee, Ali Tabatabaey

**Affiliations:** ^1^ Department of Surgery, School of Medicine Ardabil University of Medical Sciences Ardabil Iran; ^2^ Department of Pediatric Surgery, Faculty of Medicine Mashhad University of Medical Sciences Mashhad Iran; ^3^ Division of Emergency Medicine University of Toronto Temerty Faculty of Medicine Toronto Ontario Canada

**Keywords:** hypertrophic pyloric stenosis, pyloromyotomy, situs inversus totalis, vomiting

## Abstract

The synchronicity of situs inversus totalis (SIT) and infantile hypertrophic pyloric stenosis (IHPS) is rare. We have reported a case of this concurrency in a 3‐day‐old newborn with classic symptoms.

## Graphical abstract

Even non‐bilious vomiting can signify a severe underlying condition in newborns and demands careful evaluation.

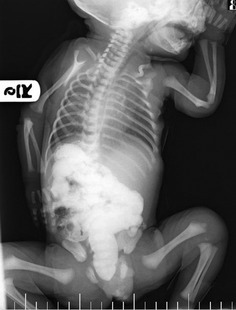

## 1 INTRODUCTION

The synchronicity of SIT and IHPS in infants is a rare phenomenon that has only been reported in two cases in the literature.^1^, ^2^ SIT occurs when the major visceral organs are completely mirror‐imaged, arranged along a left–right axis. The prevalence of this rare congenital condition is estimated at 0.1—0.6 per 10,000 live births.^3^, ^4^ SIT does not usually cause complications and may be incidentally diagnosed in later decades of life. IHPS is a less rare condition in infants characterized by an acquired narrowing of the pylorus. A near‐complete obstruction of the pylorus is created by progressive hypertrophy of the pyloric muscle. The typical symptom is postprandial forceful projectile vomiting leading to dehydration and a hypochloremic metabolic alkalosis in infants.^5^ Its incidence is estimated between 2 and 5 per thousand live births in western countries but is less common in other parts.^6^ Epidemiologically, infants get symptomatic between 3 weeks and three months.^7^ We are reporting a case of IHPS who presented on day three of life.

## 2 CASE PRESENTATION

**FIGURE 1:**
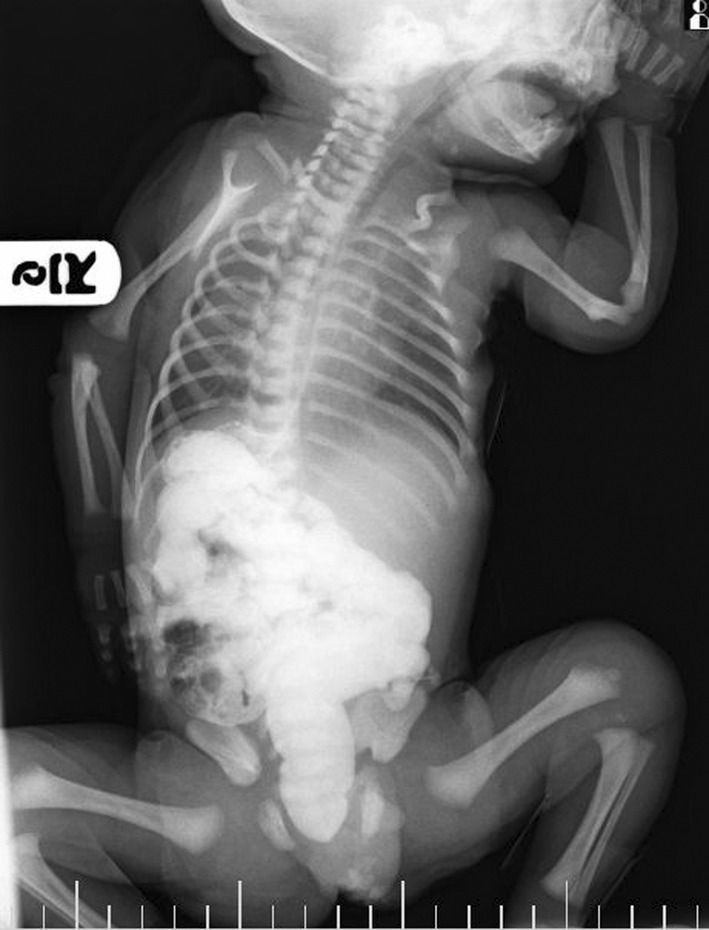
UGI barium contrast was showing situs inversus totalis

A 3‐day‐old term infant product of an uncomplicated pregnancy and normal vaginal delivery with a birthweight of 2.2 kg was referred to the pediatric surgery service on his second day of life because of non‐bilious vomiting. The vomiting was progressive, usually followed breastfeeding, and was not accompanied by abdominal distention or defecation problems. Findings on ultrasonography revealed that the pyloric canal is 22 mm in length and 4 mm in width. No other abdominal anomalies were reported. An incidental finding was the mirror displacement of the abdominal viscera. In clinical examination, the “olive sign” was absent. Preoperative echocardiography and upper gastrointestinal (UGI) barium contrast series were requested for preoperative preparation, demonstrating dextrocardia (Figure 1). Given typical imaging findings, the diagnosis of situs inversus totalis was confirmed.

Since IHPS does not usually present this early in life, an upper gastrointestinal series was also performed to rule out other possible causes. No evidence of malrotation or midgut volvulus was seen. Barium enema did not reveal evidence of intestinal atresia.

**FIGURE 2:**
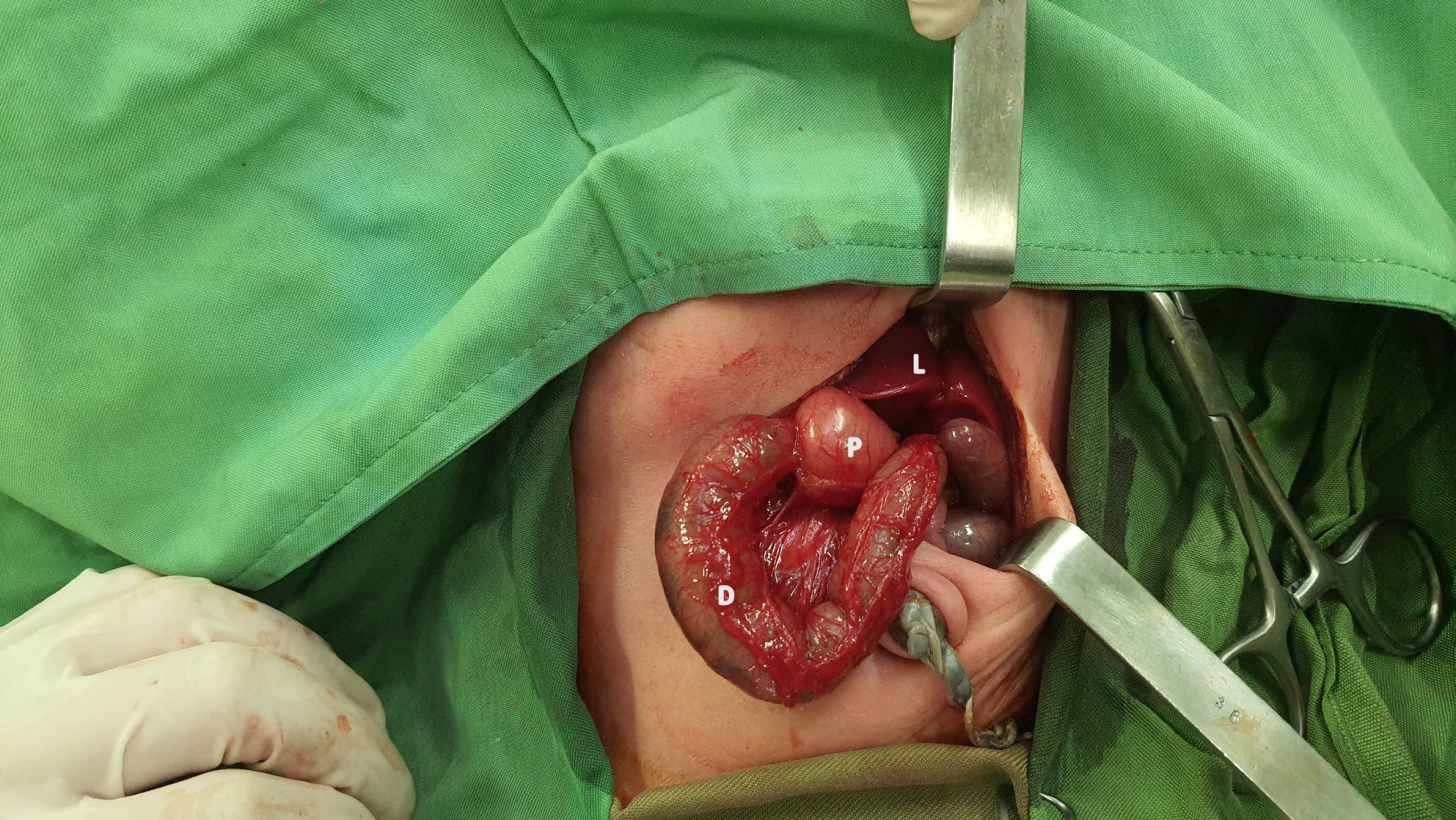
Left upper quadrant transverse incision of the liver (L) appeared in the left and hypertrophied pylorus (P) with duodenum (D) in the right

**FIGURE 3:**
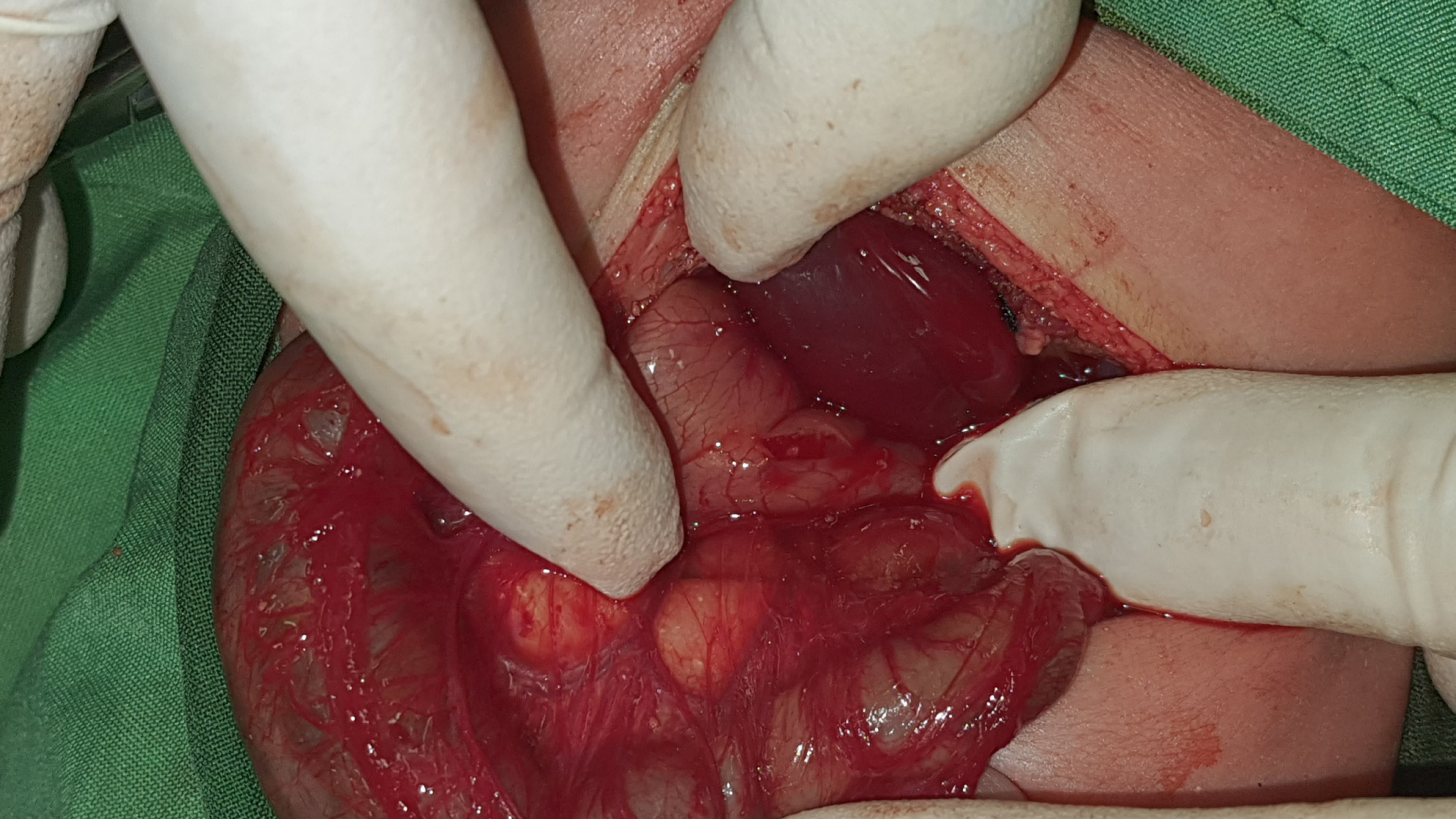
Pyloromyotomy procedure

Fluid resuscitation was continued for 24 hours to normalize acid–base disturbances, electrolytes, and bicarbonate levels. We underwent abdominal exploration through a transverse left‐leaning supraumbilical incision after stabilization. No anomaly except SIT and a thickened pylorus on the left side were detected. The thickness zone was visible in the pyloric region, compatible with the ultrasonography report (Figure 2). Classic Ramstedt pyloromyotomy was done (Figure 3). After checking for a leak by insufflation of 60 ml of air through a nasogastric tube, the pylorus was returned to the abdominal cavity. Feeding with Pedialyte was initiated four hours postoperatively. He was discharged from the hospital the following day. Postoperative and 3‐month follow‐up visits were uneventful, and he remains within the normal range on growth charts.

## 3 DISCUSSION

Situs inversus viscerum occurs when intra‐abdominal organs are mirrored inside the abdomen. This rare phenomenon has a prevalence rate of 0.1—0.6 per 10,000 live births.^1^, ^2^ Diagnosis of SIT is usually delayed from the neonatal period to infancy only after radiographic evaluation for respiratory distress or another reason.^8^ A radiographic examination reveals the stomach bubble in the right abdomen and the apex of the heart pointing to the right. In the majority of cases, any complications are not associated with SIT. However, neonatal intestinal obstruction,^9^ congenital heart defects, primary ciliary dyskinesia, renal disorders, biliary atresia, skeletal dysplasia, and mental retardation^10^ are associated with this anomaly. SIT accompanying infantile hypertrophic pyloric stenosis in an infant is a rare phenomenon reported in only 2 cases in the literature.^1^, ^2^ In infantile hypertrophic pyloric stenosis (IHPS), young infants of 3 weeks to 3 months suffer from the acquired hypertrophy of the muscular layer of the stomach's antropyloric portion, causing the obstruction. Isolated IHPS is the most common diagnosis requiring surgery.^11^ Reports estimate an incidence of 2 to 5 per thousand live births in the Western world.^12^ Male infants are more affected, with a 4:1 male‐to‐female.^13^ Risk factors mentioned in the literature include family history, gender, younger maternal age, being a first‐born infant, and maternal feeding patterns.^14^ The infants at birth appear normal but develop non‐bilious forceful projectile vomiting within the first few weeks of life.

Pyloric muscle progressively gets hypertrophied and obstructs gastric emptying. Since sometimes the emesis is infrequent, it may be misdiagnosed as gastroesophageal reflux disease (GERD). If left untreated, there is a risk of death due to dehydration, malnutrition, and hypochloraemic, hypokalaemic metabolic alkalosis. The typical examination finding is palpation of the “olive” in the epigastrium, the thickened pylorus. However, it is not uniformly present, and its recognition is hampered by the examiner's experience, presence of gastric distention, and an unsettled infant. For these reasons, ultrasonography is the investigation of choice with a sensitivity and specificity of 100% in experienced hands.^15^ In treating IHPS, the priority is fluid resuscitation and electrolytes replacement, followed by feedings cessation. The pyloromyotomy is not an emergent procedure and is considered the second line. Gastric decompression is controversial but is used in severe cases to reduce the risk of aspiration. Several surgical approaches exist, from minimally invasive laparoscopy to open pylorus access. The typical right upper quadrant transverse incision is used most commonly. In our patient, given the SIT, we performed a left upper quadrant transverse incision instead and extended it to explore the entire intra‐abdominal cavity for all possible anomalies.

## 4 CONCLUSION

Vomiting in the newborn is severe, even if it is non‐bilious and needs careful evaluation. Although IHPS is a disorder of infants generally aged three weeks to three months, it may rarely present earlier. Very rarely, as in our patient, SIT can be associated with gastrointestinal disorders such as IHPS.

## AUTHOR CONTRIBUTIONS

Khashayar Atqiaee and Ali Tabatabaey involved in planning. Khashayar Atqiaee and Ali Samady Khanghah involved in conduct. Khashayar Atqiaee involved in reporting. Ali Samady Khanghah involved in data acquisition. Ali Samady Khanghah involved in data interpretation. Ali Tabatabaey involved in manuscript preparation, editing, and review.

## FUNDING INFORMATION

The authors received no funds to conduct the study. It was self‐funded.

## CONFLICT OF INTEREST

The authors declare no competing interests relating to this original work.

## ETHICAL APPROVAL

We confirm that all named authors have read and approved the manuscript. The protection of intellectual property associated with this manuscript has been our consideration.

## CONSENT

The authors confirmed that they had gotten all proper patient written consent formats. The parents have given their consent for their images and other clinical information to be reported in the form. We informed The parents about the confidentiality of the names and initials, and efforts would be made to hide their identity.

## Data Availability

The data that support the findings of this study are available from the corresponding author upon reasonable request.
